# Candidemia in non-ICU surgical wards: Comparison with medical wards

**DOI:** 10.1371/journal.pone.0185339

**Published:** 2017-10-18

**Authors:** Antonio Vena, Emilio Bouza, Maricela Valerio, Belén Padilla, José Ramón Paño-Pardo, Mario Fernández-Ruiz, Ana Díaz Martín, Miguel Salavert, Alessandra Mularoni, Mireia Puig-Asensio, Patricia Muñoz, for the CANDIPOP Project

**Affiliations:** 1 Clinical Microbiology and Infectious Disease Division, Hospital General Universitario Gregorio Marañón, Madrid, Spain; 2 Instituto de Investigación Sanitaria Hospital Gregorio Marañón, Madrid, Spain; 3 Medicine Department, School of Medicine, Universidad Complutense de Madrid, Madrid, Spain; 4 Clinica Malattie Infettive AOU Santa Maria della Misericordia Piazzale Santa Maria della Misericordia, Udine, Italy; 5 CIBER Enfermedades Respiratorias-CIBERES (CB06/06/0058), Madrid, Spain; 6 Hospital Universitario La Paz, Madrid, Spain; 7 Hospital Universitario 12 de Octubre, Instituto de Investigación Hospital 12 de Octubre (i+12), Madrid, Spain; 8 Servicio Andaluz de Salud, UGC-SCCU, Sevilla, Andalucía, Spain; 9 Hospital Universitario y Politécnico La Fe, Valencia, Spain; 10 Istituto mediterraneo per i trapianti e terapie ad alta specializzazione ISMETT-UPMC, Palermo, Italy; 11 Hospital Universitario Vall d'Hebron, Barcelona, Spain; Louisiana State University, UNITED STATES

## Abstract

Candidemia acquired outside critical care or hematological areas has received much attention in recent years; however, data on candidemia in surgical departments are very scarce. Our objectives were to describe episodes of candidemia diagnosed in surgical wards and to compare them with episodes occurring in medical wards. We performed a post hoc analysis of a prospective, multicenter study implemented in Spain during 2010–2011 (CANDIPOP project). Of the 752 episodes of candidemia, 369 (49.1%) occurred in patients admitted to surgical wards (165, 21.9%) or medical wards (204, 27.2%). Clinical characteristics associated with surgical patients were solid tumor as underlying disease, recent surgery, indwelling CVC, and parenteral nutrition. Candidemia was more commonly related to a CVC in the surgical than in the medical wards. The CVC was removed more frequently and early management was more appropriate within 48 hours of blood sampling in the surgical patients. Overall, 30-day mortality in the surgical departments was significantly lower than in medical wards (37.7% vs. 15.8%, p<0.001). Multivariate analysis revealed admission to a surgical ward and appropriate early management of candidemia as factors independently associated with a better outcome. We found that approximately 50% of episodes of candidemia occurred in non-hematological patients outside the ICU and that clinical outcome was better in patients admitted to surgical wards than in those hospitalized in medical wards. These findings can be explained by the lower severity of underlying disease, prompt administration of antifungal therapy, and central venous catheter removal.

## Introduction

Candidemia is a leading cause of nosocomial bloodstream infections[1, 2] and has a high attributable mortality[3–6]. Most series have addressed the characteristics and management of the disease in onco-hematological[7–9] or critically ill patients[10–20], although in recent years, attention has shifted towards episodes of candidemia in internal medicine departments[21–25].

Data on candidemia in surgical departments are very scarce and only episodes occurring in specific surgical setting have been studied[26]. Furthermore, to our knowledge, candidemia in surgical wards has never been directly compared with episodes in medical wards.

We performed a sub-analysis of the CANDIPOP study in order to describe episodes of candidemia diagnosed in surgical wards, to compare them with episodes occurring in medical wards and to analyze the impact of therapeutic strategies on mortality.

## Materials and methods

### Patients’ cohort

The findings reported here comprise a sub-analysis of the multicenter Population Study on Candidemia in Spain (CANDIPOP). The period of candidemia surveillance was May 2010 through April 2011 and included all patients who developed an episode of candidemia (regardless of the hospital ward in which the infection occurred) in 29 participating hospitals, located in five of the largest municipal areas of Spain (population 9 498 980, or 20% of the Spanish population). The inclusion criteria, study population, methodology, microbiological studies, and outcomes have been extensively described elsewhere[15, 27]. Briefly, during the study period local laboratories daily identified patients and reported them to study coordinators, who collected data using a standardized case report form. Data included demographic and clinical characteristics, risk factors for candidemia, antifungal management and source control. Thirty-day follow-up outcome was recorded for each patient. Given the observational nature of the study, patients were managed according to routine clinical care.

The local institutional review boards of each participating center (Hospital General Universitario Gregorio Marañón, Madrid; Hospital Universitario La Paz, Madrid; Hospital Universitario Ramón y Cajal, Madrid; Hospital Infanta Leonor, Madrid; Hospital Universitario La Princesa, Madrid; Hospital Universitario del Niño Jesús, Madrid; Hospital Universitario “12 de Octubre,” Madrid; Hospital Clínico San Carlos, Madrid; Fundación Jiménez Díaz, Madrid; Hospital de Alcorcón, Madrid; Hospital Universitario Puerta de Hierro-Majadahonda, Madrid; Centro Nacional de Microbiología, Instituto de Salud Carlos III, Majadahonda, Madrid; Hospital Universitario Virgen Macarena, Sevilla; Hospital Universitario Virgen de Valme, Sevilla; Hospital Universitario Virgen del Rocío, Sevilla; Hospital Quirón Sagrado Corazón, Sevilla; Hospital San Juan de Dios de Aljarafe, Sevilla; Hospital Universitari La Fe, Valencia; Hospital Clínico Universitario de Valencia; Hospital Universitario Dr. Peset, Valencia; Consorcio Hospital General Universitario de Valencia; Hospital de Basurto, Bilbao; Hospital Universitario de Cruces, Bilbao; Hospital de Galdakano, Bilbao; Hospital Universitario Vall d’Hebron, Barcelona; Hospital Clínico IDIBAPS, Barcelona; Hospital Universitario de Sant Pau i Santa Creu, Barcelona; Hospital de Barcelona, Barcelona; Hospital del Mar, Barcelona; Hospital Sant Joan de Déu, Esplugues de Llobregat, Barcelona) approved this study, and written informed consent was obtained from each patient before enrollment. The IRB of the coordinating center for this study was Comité Ético de Investigación Clínica, Hospital General Universitario Gregorio Marañón.

### Definitions

Surgical patients were those admitted to a general or specialist surgical ward when the first blood sample yielding *Candida* spp. was taken. Medical patients were those admitted to an internal medicine ward or to a subspecialty ward of internal medicine at the time the positive sample was drawn.

Proven catheter-related candidemia was defined according to current guidelines [28], whereas secondary episodes required microbiological documentation of the same *Candida* species at the onset of the infection[15]. When there were no apparent infections at other sites, candidemia was classed as primary.

We defined initial therapy as the first systemic antifungal drug administered after a positive sample drawn from a peripheral vein. Neutropenia was defined as a neutrophil count ≤500/mm^3^.

Severity of illness was classified according to the Pitt score[29]. Sepsis, severe sepsis or septic shock were recorded on the day of candidaemia [30]. An episode of candidemia was defined as persistent when patients had positive follow-up blood cultures at least 48 hours after the initiation of antifungal therapy.

In order to assess the impact of therapeutic measures on outcome, antifungal therapy and removal of the central venous catheter (CVC) were evaluated according to previous definitions[15], as follows:

1. *Early antifungal treatment* was defined as *adequate* if a recommended dose of an antifungal drug was administered within 48 hours after candidemia onset and it was found to be effective by in-vitro susceptibility testing.
2. *Early CVC removal* was defined as removal of the indwelling catheter within 48 hours after the index blood sample was drawn. In patients who had multiple CVCs, removal of at least the responsible CVC within this timeframe was required.
3. *Early*, *appropriate management of candidemia* was defined as administration of one or more appropriate antifungal drugs (according to in-vitro susceptibility testing) and removal of the CVC within 48 hours of blood sampling. The primary outcome of the study was the 30-day mortality rate.

### Data collection

Data were prospectively recorded on a standardized case report form that included demographics, predisposing risk factors within the preceding 30 days, and clinical management. Patients were followed for 30 days and managed according to routine clinical care. No specific recommendations were provided. Laboratories were regularly audited to ensure that all cases were reported.

### Microbiological methods

Blood cultures were processed at each participating hospital. Species were identified using routine methods at the local laboratories and *Candida* isolates were forwarded to the Mycology Reference Laboratory (MRL), National Center for Microbiology (Madrid, Spain), where species identification was confirmed by sequencing the internal transcribed spacer (ITS) regions from ribosomal DNA. ITS1 and ITS2 regions were directly amplified by PCR from yeast suspensions and sequenced using universal primers. Antifungal susceptibility testing was performed according to the EUCAST- method [31]. *C*. *parapsilosis* ATCC 22019 and *C*. *krusei* ATCC 6258 were used as quality control strains for antifungal drug susceptibility testing.

### Statistical analysis

Descriptive statistics were used to summarize the data. Quantitative variables are reported as median and interquartile range (IQR), and categorical variables as counts (%). The chi-square test or Fisher exact tests were used to compare the distribution of categorical variables, including the clinical characteristics of medical and surgical patients and the association between individual risk factors and mortality rate. The *t* test or Mann-Whitney test was used to compare quantitative variables. Statistical significance was set at p<0.05. Only the first episode of candidemia recorded for an individual patient was considered in the analysis of mortality. The Kaplan-Meier curve was constructed to show the relationship between therapeutic strategies and 30-day survival. Factors that occurred in >10% of patients in the univariate analysis were evaluated in a logistic regression model to test their relationship with mortality. The statistical analyses were performed using Microsoft SPSS PC+, version 15.0 (SPSS, Chicago, Illinois, USA).

## Results

During the 1-year study period, a total of 752 episodes of *Candida* BSI were included in the CANDIPOP study. Of these, 273 (36.3%) were in the ICU, 37 (4.9%) in the emergency department, 36 (4.8%) in hematology wards, 24 (3.2%) pediatric units, and 13 (1.7%) in other wards. The remaining 369 cases (49.1%) occurred in adult patients admitted to either surgical (165 episodes, 21.9%) or medical wards (204 episodes, 27.2%) and are the subject of the present study.

### Clinical characteristics of patients admitted to surgical wards

The demographics and clinical features of the 165 patients hospitalized in a surgical ward at the time of their episode are shown in Table 1. Mean age was 65.2 years (range 16–94 years), and 103 patients were men (62%). Most candidemia episodes in surgical wards were diagnosed in a general surgery department (104, 63.0%), followed by urology (13.3%), vascular surgery (7.3%), neurosurgery (5.4%), and traumatology (4.2%).

**Table 1:** Comparison of the main demographic and clinical characteristics between patients admitted to surgical and medical wards.

VARIABLE	Surgical N = 165 (%)	Medical N = 204 (%)	p-*value*
**Demographics**			
Age, years	65.2 ± 16.6	66.9 ± 16.2	0.30
Sex, male	103 (62.4)	117 (57.4)	0.32
Days of hospital stay until *Candida* BSI	26 (14–53)	19 (9–39)	**0.003**
**Comorbidities**			
Solid tumor	85 (52.1)	83 (41.3)	0.04
Solid organ transplantation	5 (3.0)	10 (4.9)	0.43
HIV/AIDS	1 (0.6)	8 (3.9)	0.06
Cardiac disease	55 (33.3)	73 (35.8)	0.62
Pulmonary disease	30 (18.2)	55 (27.0)	**0.04**
Liver disease	15 (9.1)	49 (24.0)	**<0.001**
Renal insufficiency	26 (15.8)	73 (35.8)	**<0.001**
Diabetes mellitus	34 (20.6)	64 (31.4)	**0.02**
Neurologic disease	27 (16.4)	59 (28.9)	**0.005**
Autoimmune disease	5 (3.0)	8 (3.9)	0.78
**Previous antifungal treatment**	31 (18.8)	33 (16.2)	0.58
**Risk factors for candidemia**			
Previous antibiotic therapy	156 (94.5)	188 (92.2)	0.69
Previous corticosteroid therapy	31 (18.8)	64 (31.4)	**0.004**
Immunosuppressive therapy	19 (11.5)	62 (30.4)	**<0.001**
Central venous catheter	127 (77.0)	126 (61.8)	**0.02**
Surgery (all types <3 months)	138 (83.6)	56 (27.5)	**<0.001**
Abdominal surgery	93 (56.4)	24 (11.8)	**<0.001**
Neutropenia	1 (0.6)	5 (2.5)	0.23
Mucositis at diagnosis	2 (1.2)	14 (6.9)	0.03
TPN during candidemia	94 (57.0)	71 (34.8)	**<0.001**
**Source of infection**			
Primary	69 (41.8)	131 (64.2)	**<0.001**
Central venous catheter	71 (43.0)	58 (28.4)	**0.004**
Abdomen	9 (5.5)	4 (2.0)	0.09
Urinary tract	15 (9.1)	11 (5.4)	0.22
**Pitt score**	1 (0–2)	1 (0–2.75)	**0.04**
**Clinical features**			
Sepsis	137 (83)	161 (78.9)	0.35
Severe sepsis or septic shock at onset	28 (17.0)	43 (21.1)	0.35
ICU admission	16 (9.7)	16 (7.8)	0.52
Concomitant bacteremia	29 (17.6)	31 (15.2)	0.57
**Spread to other organs**			
Spleen	2 (1.2)	1 (0.5)	0.19
Eyes	6 (3.6)	2 (1.0)	0.14
Heart	4 (2.4)	4 (2.0)	0.84
Other*	6 (3.8)	3 (1.5)	**0.04**
***Candida* species**			
*C*. *albicans*	84 (50.9)	89 (43.6)	0.17
*C*. *parapsilosis*	36 (21.8)	43 (21.1)	0.89
*C*. *glabrata*	24 (14.5)	28 (13.7)	0.88
*C*. *tropicalis*	12 (7.3)	21 (10.3)	0.36
*C*. *krusei*	5 (3.0)	6 (2.9)	1
*C*. *guilliermondii*	2 (1.2)	3 (1.5)	1
Other	2 (1.2)	14 (6.9)	**0.009**

**ICU** intensive care unit **TPN** total parenteral nutrition:

*Other: central nervous system, lungs, and septic thrombophlebitis

The most common underlying condition was solid tumor (52.1%) followed by cardiac disease (55 patients, 33.3%). Almost all patients had recently undergone surgery (within the previous 3 months). A CVC was in place in 127 patients (77%), and 94 (57%) were receiving total parenteral nutrition at the time of their episode. The most prevalent source of infection was the CVC (43%); only 9 patients (5.5%) had an abdominal infection. Candidemia was managed early and appropriately within the first 48 hours after blood sample collection in 39.4% of patients. Fluconazole was the preferred therapy in most cases, and 30-day mortality was 15.8%.

### Comparison between medical and surgical patients

The main demographic characteristic and risk factors for candidemia in medical and surgical patients are compared in Table 1. Age and sex were similar between groups.

In the surgical wards, candidemia affected significantly more patients with solid tumors (52.1% vs 41.3%, p = 0.04), and significantly fewer with diabetes (16.4% vs 28.9%, p = 0.02), pulmonary disease (18.2% vs 27.0%, p = 0.04), liver disease (15.0% vs 24.0%, p<0.001), neurological disease (16.4% vs 28.9%, p = 0.005), and renal insufficiency (15.8% vs 35.8, p<0.001).

Risk factors for candidemia were also different between the two groups. In the surgical wards, the main factors were surgical intervention (83.6% vs 27.5%, p<0.001), presence of a long term CVC (77% vs 61.8%, p = 0.02), and administration of total parenteral nutrition (57% vs 34.8%, p<0.001); whereas in the medical wards the most frequent factors were previous treatment with corticosteroids (18.8% vs 31.4%, p = 0.004) and other immunosuppressive agents (11.5% vs 30.1%, p<0.001).

Median time to onset of candidemia after admission was significantly longer for surgical patients (26 days) than for medical patients (19 days, p = 0.003). As for source of infection, surgical patients were more likely to have CVC-related candidemia (43% vs 28.4%, p = 0.004) and an intra-abdominal origin (5.5% vs 2%, p = 0.09), whereas medical patients were likely to have candidemia of primary origin (41.8% vs 64.2%, p<0.001). No further significant differences were found between the groups regarding clinical manifestations of the diseases, species distribution, or severity of illness except for the median Pitt score, which was lower in surgical patients than in medical patients (p = 0.004).

In terms of antifungal treatment (Table 2), the only significant difference between the surgical and medical wards was that a higher percentage of medical patients never received an antifungal drug (7.3% vs 15.2%, *p* = 0.02), because the diagnosis was made peri- or post-mortem.

**Table 2:** Therapeutic measures and outcome of patients admitted to medical vs surgical wards.

VARIABLE	Surgical N = 165 (%)	Medical N = 204 (%)	p-*value*
**Initial antifungal agents**			
Azoles	104 (63.0)	113 (55.4)	0.16
Echinocandins	36 (21.8)	41 (20.1)	0.70
Liposomal amphotericin B	3 (1.8)	6 (2.9)	0.73
Combination	10 (6.1)	13 (6.4)	1
No antifungal therapy	12 (7.3)	31 (15.2)	**0.02**
**Fluconazole-non-susceptible strains**	4/159 (2.5)	13/188 (6.9)	0.07
**Length of antifungal treatment** (mean ± SD), **days**	7.8 ± 7.6	9.4 ± 12.0	0.14
**Adequate empirical antifungal therapy** (before positive blood culture)	6 (3.6)	4 (2.0)	0.35
**Appropriate early antifungal therapy** (within 48 h of blood culture collection)	69 (41.8)	69 (33.8)	0.11
**Early central venous catheter removal** (within 48 h of blood culture collection)	96 (58.2)	76 (37.4)	**<0.001**
**Early adequate candidemia management** (within 48 h of blood culture collection)	65 (39.4)	58 (28.4)	**0.035**
**Persistent candidemia**	42/121 (34.7)	36/121 (29.8)	0.49
**Median time to death**	17.5 (7–36)	12 (5–31)	0.37
**30-day mortality**	26 (15.8)	77 (37.7)	**<0.001**

Of note, early removal of the CVC and appropriate early management of candidemia were more common in patients in surgical wards than in medical wards (58.2% vs 37.4% [p<0.001] and 39.4% vs 28.4% [p = 0.035], respectively). As for other indicators of quality of care, control blood cultures were more frequently performed in surgical wards (73.3% vs 59.3%. p = 0.035), although the percentage of patients with persistent fungemia did not differ between the groups.

The 30-day mortality rate was 15.8% in the surgical wards and 37.7% in the medical wards (p<0.001).

### Outcome and risks factors for mortality

As shown in Table 3, the variables associated with higher 30-day mortality in the univariate analysis were older age, liver disease, previous antifungal therapy, neutropenia, primary source of infection, higher Pitt score, severe sepsis or septic shock at onset and no treatment for candidemia. The variables associated with lower mortality were previous surgery, CVC-related candidemia, azole therapy, early adequate management of candidemia, and admission to a surgical ward.

**Table 3:** Univariate analysis of factors associated with 30-day mortality in medical and surgical wards.

VARIABLE	Alive N = 266 (%)	Died N = 103 (%)	p-*value*
**Demographics**			
Age, years	65.1±15.9	68.8±17.3	**0.05**
Sex, male	157 (59)	63 (61.2)	0.72
**Hospital admission at the time of the candidemia episode**			
Medical ward	127 (47.7)	77 (74.8)	**<0.001**
Surgical ward	139 (52.3)	26 (25.2)	
**Comorbidities**			
Solid tumor	115 (43.2)	53 (51.5)	0.16
Solid organ transplantation	12 (4.5)	3 (2.9)	0.35
HIV/AIDS	4 (1.5)	5 (4.9)	0.12
Cardiac disease	88 (33.1)	40 (38.8)	0.33
Pulmonary disease	57 (21.4)	28 (27.2)	0.27
Liver disease	36 (13.5)	28 (27.5)	0.003
Renal insufficiency	64 (24.1)	35 (34)	0.06
Diabetes mellitus	66 (24.8)	32 (31.1)	0.23
Neurologic disease	61 (22.9)	25 (24.3)	0.78
Autoimmune disease	11 (4.1)	2 (1.9)	0.59
**Previous antifungal treatment**	39 (14.7)	25 (24.3)	**0.03**
**Risk factors for candidemia**			
Previous antibiotic therapy	251 (94.4)	93 (90.3)	0.17
Previous corticosteroid therapy	62 (23.3)	33 (32.0)	0.11
Immunosuppressive therapy	53 (19.9)	28 (27.2)	0.16
Central venous catheter	185 (69.5)	68 (66.0)	0.53
Surgery (all types <3 months)	159 (59.8)	35 (34.0)	**<0.001**
Abdominal surgery	97 (36.5)	20 (19.4)	**0.02**
Neutropenia	1 (0.4)	5 (4.9)	**<0.007**
Mucositis at diagnosis	8 (3.0)	8 (7.8)	0.08
TPN during candidemia	123 (46.2)	42 (40.8)	0.35
**Source of infection**			
Primary	129 (48.5)	71 (68.9)	**<0.001**
Central Venous Catheter	105 (39.5)	24 (23.3)	**0.003**
Abdomen	22 (8.3)	4 (3.9)	0.17
Urinary tract	9 (3.4)	4 (3.9)	0.76
**Pitt score**	1 (0–2)	2 (1–4)	**<0.001**
**Clinical features**			
Sepsis	230 (86.5)	68 (66.0)	**<0.001**
Severe sepsis or septic shock at onset	36 (13.5)	35 (34.0)	**<0.001**
ICU admission	22 (8.3)	10 (9.7)	0.68
Concomitant bacteremia	38 (14.3)	22 (21.4)	0.11
Persistent candidemia	63 (30.6)	15 (41.7)	0.24
**Spread to other organs**			
Spleen	3 (1.1)	0	0.56
Eyes	7 (2.1)	1 (1)	0.70
Heart	5 (1.9)	2 (1.9)	1
Other*	7 (2.6)	2 (1.9)	1
***Candida* species**			
*C*. *albicans*	141 (53)	55 (53.4)	1
*C*. *parapsilosis*	60 (22.6)	19 (18.4)	0.47
*C*. *glabrata*	39 (14.7)	13 (12.6)	0.73
*C*. *tropicalis*	24 (9)	9 (8.7)	1
*C*. *krusei*	6 (2.3)	5 (4.9)	0.18
*C*. *guilliermondii*	3 (1.1)	2 (1.1)	0.62
Other	9 (3.4)	7 (6.8)	0.16
**Fluconazole-non-susceptible strains**	10/254 (3.9)	7/93 (7.5)	0.17
**Empirical antifungal therapy (before positive blood culture)**	42 (15.8)	17 (16.5)	0.87
**Appropriate early antifungal therapy**	175 (65.8)	48 (46.6)	**0.001**
**Initial antifungal agents**			
Azoles	172 (64.7)	45 (43.7)	**<0.001**
Echinocandins	56 (21.1)	21 (20.4)	1
Liposomal amphotericin B	7 (2.6)	2 (1.9)	1
Combination	18 (6.8)	5 (4.9)	0.63
No antifungal therapy	13 (4.9)	30 (29.1)	**<0.001**
**Early CVC removal**	146 (54.9)	26 (25.5)	**<0.001**
**Appropriate early management of candidemia**	107 (40.2)	16 (15.5)	**<0.001**

**CVC** central venous catheter; **ICU** intensive care unit **TPN** total parenteral nutrition;

*Other: central nervous system, lungs, and septic thrombophlebitis

Multivariate analysis (Table 4) showed that the variables associated with mortality were liver disease (OR, 2.68; 95%CI, 1.29–5.55; p = 0.008), neutropenia (OR, 14.80; 95%CI, 1.55–140.98; p = 0.01), higher Pitt score (OR, 1.37; 95%CI, 1.17–1.67; p<0.001), and absence of antifungal therapy (OR, 5.64; 95%CI, 1.94–16.35; p = 0.001). By contrast, the variables that were independently associated with a better outcome were admission to a surgical ward (OR, 0.51; 95%CI, 0.28–0.92; p = 0.03) and appropriate early management of candidemia (OR, 0.27; 95%CI, 0.13–0.55; p = 0.001).

**Table 4:** Multivariate logistic regression analysis of prognostic factors associated with 30-day mortality.

VARIABLE	OR	95% CI	p-*value*
**Neutropenia**	**14.80**	**1.55–140.98**	**0.01**
**No antifungal therapy**	**5.64**	**1.94–16.35**	**0.001**
**Liver disease**	**2.68**	**1.29–5.55**	**0.008**
**Pitt score**	**1.37**	**1.17–1.67**	**<0.001**
Central venous catheter-related candidemia	1.21	0.41–3.57	0.71
Severe sepsis or septic shock	0.69	0.20–2.33	0.69
Age, years	1.01	0.99–1.03	0.15
Early central venous catheter removal	0.61	0.25–1.41	0.26
**Appropriate early candidemia management**	**0.27**	**0.13–0.55**	**<0.001**
**Surgical ward**	**0.05**	**0.28–0.92**	**0.03**

## Discussion

Our findings suggest that approximately 50% of episodes of candidemia occur in non-hematological patients and outside the ICU and that patients admitted to surgical wards have a better clinical outcome than those hospitalized in medical wards, mainly because of the lower severity of the underlying disease and, prompt administration of antifungal therapy, and CVC removal.

Candidemia in non-ICU, non-hematological patients has received little attention in the literature. However, in a recent paper by Bassetti et al.[21–23, 25], almost 75% of candidemic patients were hospitalized in surgical wards or internal medicine wards. Other studies have reported that 60–75% of episodes occur in non-ICU, non-hematological patients[21–23, 25]. Our report, which is one of the largest contemporary epidemiological survey of candidemia, shows that candidemia in non-ICU, non-hematological patients is a major problem, accounting for almost 50% of candidemic episodes diagnosed in Spain. Of those episodes, half occurred in surgical wards and half in internal medicine wards. The circumstances responsible for the high prevalence of candidemia in these "non-classic" settings seem to be multifactorial, including rapid changes in medical practice, ageing of the population, the increasing number of patients with CVC, and the increased number of immunocompromised patients with more complex underlying disease.

Interestingly, when major risk factors for candidemia in adult patients were analyzed[11, 32–34], significant differences were observed between surgical and medical patients. Compared with medical cases, surgical patients were much more likely to have undergone a previous surgical intervention or to have a long-term CVC in place at the time of candidemia. On the other hand, medical patients were more likely to have received treatment with corticosteroids and other immunosuppressive agents. These results suggest a different pathogenesis for candidemia between patients admitted to surgical wards and patients admitted to medical wards. Given that the sites most frequently colonized by yeasts are the gastrointestinal tract and skin[35, 36], surgical patients may have acquired fungemia because of rupture of the gastrointestinal barrier during abdominal surgery [37] or due to total parenteral nutrition or skin contamination at vascular insertion sites. Thus, attempts aimed at implementing adequate surgical procedures and optimal CVC care may have a higher impact on reducing episodes of *Candida* BSI in surgical departments. Conversely, in patients admitted to internal medicine wards, candidemia seems to be related to more frequent immune system dysfunction as a result of therapy with corticosteroids or other immunosuppressive agents. Corticosteroids are known to inhibit neutrophil ingestion and killing of *Candida* [38] and to promote microorganism translocation [39]. Therefore, since immunosuppressive treatment seems to play a critical role in the development of candidemia in the medical setting, physicians should anticipate the risk of infections and use the lowest possible dose of immunosuppressive drugs for the shortest possible time.

As for the etiology of candidemia, non-hematological patients outside the ICU present the typical distribution of *Candida* species in our geographical area, where *C*. *albicans* is predominant, followed by *C*. *parapsilosis* [21, 40], with a low frequency of fluconazole-non-susceptible strains, which in our study was only 4.6%. This finding has important implications for the selection of empirical antifungal therapy among non-hematological candidemic patients hospitalized outside ICUs.

An interesting finding in our study was the lower mortality rate in patients with candidemia hospitalized in surgical wards than in internal medicine wards. The overall mortality in patients with candidemia reported in the literature [21–23, 25] ranges from 12.5% to 29.7% for those hospitalized in surgical wards [23, 25], and from 39% to 66% for those hospitalized in internal medicine wards [23, 24]. In our series, overall 30-day mortality was 15.8% and 37.7% in surgical and internal medicine wards, respectively. To date, no in-depth analysis of the prognostic factors of surgical patients with candidemia has been performed.

Our initial hypothesis was that the lower mortality rate associated with candidemia in surgical wards probably reflects differences in the severity of underlying diseases. Indeed, medical patients were sicker and had at least one severe underlying disease such as liver disease, renal failure, and diabetes.

Nonetheless, as recently highlighted in multicenter study by Luzzati et al [41], the difference in outcome could also be related to the low suspicion of fungal infection in medical wards, with a longer time required to confirm a diagnosis. Indeed, prompt antifungal therapy is one the most important factors influencing outcome in candidemia, so even a short delay can be associated with a worse outcome [17, 42–44].

Although we do not have sufficient data, it seems that infectious disease specialists, who are more frequently consulted in surgical wards, could also have an impact on the better prognosis of surgical patients. Management of candidemia is clinically challenging, and previous studies showed that involvement of an infectious diseases consultant was associated with better management and even better prognosis [45, 46]. In our series, all indicators of quality of care (ie, follow-up blood sample collection, early antifungal therapy, catheter withdrawal) indicate that more patients in surgical than in medical wards were likely managed with an infectious diseases specialist, thus partially explaining the better prognosis observed in surgical patients. However, more studies are needed to clarify this aspect.

In the present study, we did not observe differences in mortality when echinocandin regimen were administered. Likewise, also other studies of candidemia did not find any association between outcome and treatment with azoles or echinocandins [41, 47], probably reflecting the bias of use echinocandins in the more severely ill patients. Despite this, the percentage of patients hospitalized in internal medicine wards who did not receive antifungal therapy was higher than that of patients admitted to surgical wards (15.2% vs. 7.3%), because blood cultures were observed to be positive late in the disease course. New diagnostic strategies investigating the role of serological biomarkers such as β-D-glucan [48, 49] should be applied in order to identify the medical and surgical patients at the highest risk of candidemia.

Besides adequate antifungal therapy, removal of the CVC within the first 48 hours after onset, is considered the most important determinant of outcome [50, 51]. Consistent with previous findings [43, 52], we also observed that appropriate early management of candidemia (antifungals and CVC removal within 48 hours) was the most important protective factor against mortality and was less commonly present in internal medicine wards. This observation could reflect a bias towards more catheter removal in patients who were less ill. Indeed, compared with patients hospitalized in internal medicine wards, surgical patients had a lower Pitt score and were less frequently affected by other comorbidities. Moreover, it must be acknowledged that CVC as the source of candidemia was more common among patients hospitalized in surgical wards. It has yet to be established whether there is an association between catheter removal and better outcome when the source of candidemia is not the CVC [52]. Although further evidence is needed to resolve this controversial issue, our findings indicate that the CVC should always be removed in medical and surgical patients, even when sources other than the CVC are suspected.

Our study is subject to a series of limitations. First, we did not have data regarding the overall population hospitalized in the internal medicine and surgical wards of the participating hospitals. Consequently, we were not able to calculate the incidence of candidemia in our study populations. Second, although the CANDIPOP study is a multicenter study including a large number of patients, the generalizability of the observations may be limited by differences in *Candida* epidemiology between geographical areas or by differences in medical practice or health system organization. Nevertheless, these data are important, because they reflect the most significant and robust experience of surgical patients developing candidemia in a large group of centers.

In conclusion, we found that approximately 50% of candidemic episodes occurred in non-hematological patients outside the intensive care unit and that clinical outcome was better in patients admitted to surgical wards than in those hospitalized in medical wards. These findings could be explained by the lower severity of underlying disease, prompt administration of antifungal therapy, and catheter removal.
